# Glassy Microspheres for Energy Applications

**DOI:** 10.3390/mi9080379

**Published:** 2018-07-30

**Authors:** Giancarlo C. Righini

**Affiliations:** 1 Enrico Fermi Centre, 00184 Roma, Italy; giancarlo.righini@centrofermi.it; 2 Nello Carrara Institute of Applied Physics (IFAC CNR), 50019 Sesto Fiorentino, Italy

**Keywords:** microspheres, microdevices, glass, polymers, solar energy, nuclear fusion, thermal insulation

## Abstract

Microspheres made of glass, polymer, or crystal material have been largely used in many application areas, extending from paints to lubricants, to cosmetics, biomedicine, optics and photonics, just to mention a few. Here the focus is on the applications of glassy microspheres in the field of energy, namely covering issues related to their use in solar cells, in hydrogen storage, in nuclear fusion, but also as high-temperature insulators or proppants for shale oil and gas recovery. An overview is provided of the fabrication techniques of bulk and hollow microspheres, as well as of the excellent results made possible by the peculiar properties of microspheres. Considerations about their commercial relevance are also added.

## 1. Introduction

**Figure 1:**
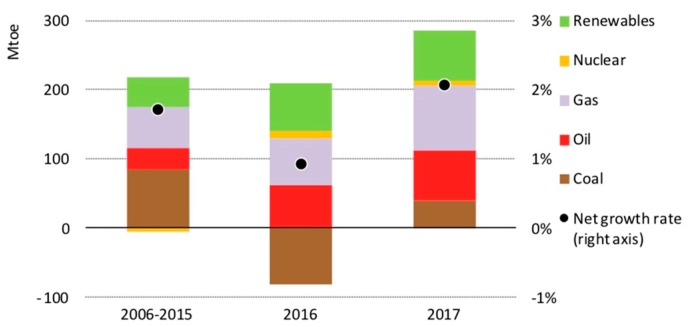
Average annual growth of the global energy demand (GED) by fuel. Reproduced from the IEA report [1].

Global energy demand (GED) keeps growing, boosted by a generally strong economic growth. According to the International Energy Agency (IEA), GED grew by 2.1% in 2017, more than twice the 2016 rate; accordingly, global energy-related carbon dioxide emissions increased by 1.4% in 2017, after three years of remaining flat [1]. Still, over 70% of GED growth was met by oil, natural gas and coal; renewable energies, however, exhibited in 2017 the highest growth rate of any energy source. Figure 1 shows the GED average annual growth for the different fuels; the y-axis on the right indicates the net growth rate, while the y-axis on the left reports the energy growth in million tons of oil equivalent (Mtoe). The overall GED in 2017 reached an estimated 14,050 Mtoe.

Continuous advancements in technology are necessary to improve production efficiency, energy security, and—last but not least—environment quality, while maintaining economic competitiveness. A not negligible contribution to some of these goals may be provided by a very simple type of microdevices, namely the microspheres, which can be either solid or hollow, the latter also known as microbubbles. In the following, the term microsphere or microbead will be used when referring to the solid object, and the term hollow microsphere or microbubble for the other type. Thanks to their physical and chemical properties, which include light weight, low thermal conductivity, resistance to compressive stress, and the fact of being almost chemically inert, microspheres and microbubbles (Ms&Mb) have been widely used in pharmaceutical, food, cosmetic, chemical, transportation and construction industrial sectors. Staying more on the research side, Ms&Mb have found advanced applications in optics and photonics; their use as whispering gallery mode (WGM) resonators opened the way to the development of several high-performance lasing and sensing micro devices [2,3]; the search for more compact and robust structures, especially in the biosensing field, is one of the current R&D trends.

Here, the analysis is limited to spherical particles in the micrometer range, which is approximately 0.1 to 1000 μm, or, in other words, from hundreds of nanometers to one millimeter. At the upper end of this range one encounters microbeads which find application in optics as microlenses, e.g., for fiber-to-fiber coupling, while at the lower end one enters into the nanotechnologies, where nanospheres and nanobubbles find several applications in photonics, catalysis, nanoreactors, drug delivery systems. An overview of the fundamentals and the applications of both glass microspheres and glass nanospheres is presented in a forthcoming book [4].

The aim of this article is to provide an overview of the Ms&Mb applications in the energy field, which can be either indirect (solar cells, thermal insulation, low-density drilling fluid for oil and gas extraction, ultra-low-density proppants for shale oil and gas recovery) or direct (in hydrogen storage and in nuclear fusion targets). A short description of the fabrication processes in the laboratory or at an industrial level will be provided, too.

## 2. Materials and Fabrication Methods

Here only microspheres and microbubbles made in amorphous materials, namely in oxide or chalcogenide glasses and in amorphous polymers, will be considered. For the sake of completeness, however, it should be noted that many other materials, either natural or synthetic, can be used to fabricate Ms&Mb for different applications. A few examples include stainless steel microspheres (for conductive spacers, shock absorption, and micromotor bearings [5]); metallic nickel hollow microspheres (enhanced magnetic properties; Ni/Pt bimetallic microbubbles have potential applications in portable hydrogen generation systems, due to catalytic properties [6]); single-crystal ferrite microspheres (for applications not only as magnetic materials but also in ferrofluid technology and in biomedical fields, e.g., biomolecular separations, cancer diagnosis and treatment, magnetic resonance imaging [7]); single-crystal semiconductor microspheres (for active WGM resonators [8]); ceramic ZrO_2_ hollow microspheres (for thermal applications) [9]. Glass, polymer, ceramic, metal solid and hollow microspheres are commercially available; there is a wide choice of quality, sphericity (Sphericity was defined in 1935 by the geologist H. Wadell, with reference to quartz particles (*J. Geology* 1935, *43*, 250) as the ratio of the surface area of a sphere (with the same volume as the given particle) to the surface area of the particle), uniformity, particle size and particle size distribution, to allow the optimal choice for each unique application.

### 2.1. Oxide Glass Microspheres

**Figure 2:**
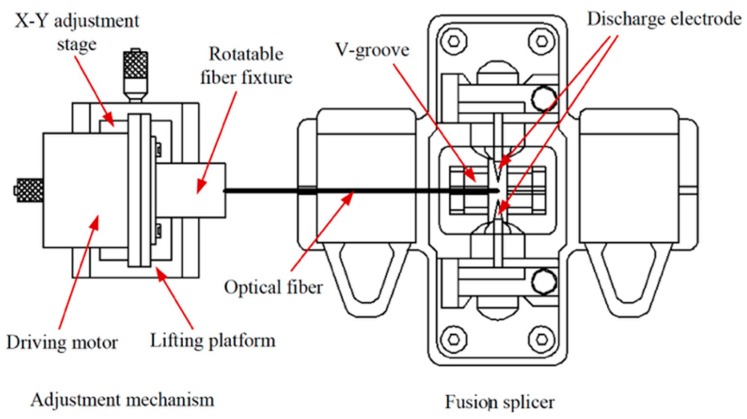
Schematic drawing of the apparatus to produce an integrated optical fiber microsphere in a fusion splicer. Reproduced from [13] under Creative Commons license.

Ms&Mb made in pure silica or multi-oxide glass are the most widely used type in research and industrial/commercial applications. At the laboratory level, when a single microsphere is needed, for instance to exploit the characteristics of a discrete WGM resonator [4] or to implement a micro/nano Coordinate Measuring Machine (CMM) probe [10], the fabrication technique may be quite a handcrafted work, requiring the care of a skilled technician. The most common method of fabrication of a high-quality microsphere, in fact, is based on the melting of the tip of a standard optical communication fiber and relies on surface tension’s effect to obtain an almost perfect spherical shape. The heating source may be a simple oxygen/butane (or similar) torch, or a high-power laser (especially CO_2_ laser), or an electric arc (such as the one produced in a commercial fiber splicer) [4,10,11,12]. For the fibers drawn from multi-oxide glasses, which have a melting point lower than pure silica, a simple resistive microheater may be sufficient [12]. The use of a commercial or modified optical fiber fusion splicer (e.g., FITEL S182K, Furukawa, Tokyo, Japan) allows a very good control of the process; a cleaved tip of the fiber is inserted in one arm of the splicer and a series of arcs are then produced. The tip partially melts, and the surface tension forces produce the spherical shape. Figure 2 presents a schematic diagram of the experimental apparatus; the heat generated from the electrode discharge can produce a temperature of around 2000 °C, which is sufficient to melt pure silica.

**Figure 3:**
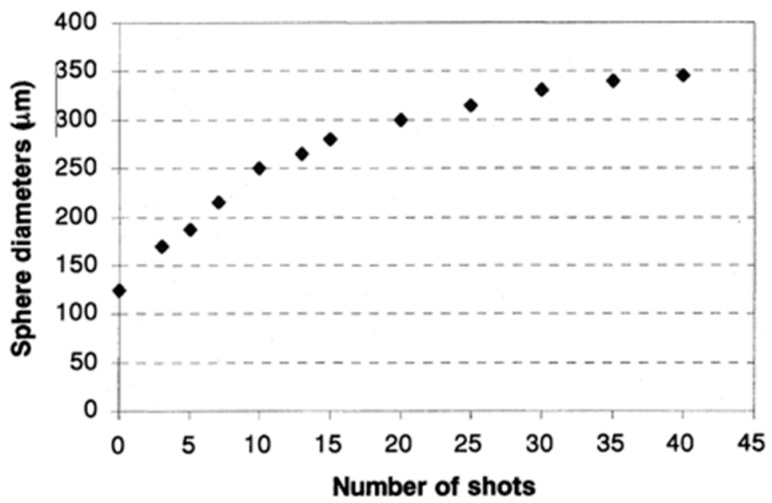
Size of the microspheres produced at the tip of a standard 125 μm telecom fiber, as a function of the arc shots in a fiber fusion splicer. Reproduced from [15].

Using this type of apparatus, Yu et al. reported the fabrication of integrated optical fiber microspheres with a diameter smaller than 100 μm, exhibiting 2D roundness error less than 0.70 μm and true sphericity of about 0.5 μm [13]. Such results were achieved by using a fiber tapering technique and a statistical process optimization method (Taguchi method) [14]. The authors here define the true sphericity as a radius difference between a perfect sphere and the 3D fitting surface profile, obtained by a fitting numerical procedure from the photograph of the 2D cross-section of the microsphere under analysis [13]. If the fiber is not tapered in advance, the size of the sphere is larger than 125 μm, namely the cladding diameter of the standard telecom SMF-28 SM silica fiber, and it increases with the number of electric arc shots until approaching saturation at a diameter of about 350 μm, as shown in Figure 3 [15].

With the previous methods, only one microsphere can be produced at a time and the size is mostly determined by the fiber size; moreover, the microsphere remains integral with the fiber stem, which is very useful in some applications but may be disadvantageous in others. When a discrete particle is needed, it is convenient to start producing a fine glass powder and then melt it. This approach also permits making microspheres from any oxide glass. As an example, one can crush the glass into particles with sizes ranging from 10 to 100 μm, and single microspheres can be obtained by the localized-laser-heating (LLH) technique, where a cw Ti: sapphire laser with typical power 200 mW at λ = 810 nm is used [16]. Other options include using a microwave plasma torch (the glass grains are dropped through it and the spherical particles are collected at the bottom) [17], or—if starting from the raw components - melting the glass components in a furnace and dropping the viscous glass onto a spinning plate [18]. A disadvantage of these techniques is that one obtains several free spheres with a rather large size distribution; it is, therefore, required to sort the produced spheres by size, while also checking their surface quality. 

A similar situation exists for hollow microspheres (aka microbubbles and microballoons). In the laboratory, a usual objective is to fabricate the microbubble integral to a capillary, to form a system of a WGM resonator with integrated microfluidics, which is very convenient for biomedical applications [19,20]. A common technique consists in using a slightly pressurized silica capillary and melting a small volume of it by using a CO_2_ laser or an electric arc discharge. Single- and double-pass structures, i.e., spherical shells with one or two openings, can be made [20]. For commercial applications, instead, discrete microballoons are required, and appropriate fabrication methods have been developed over the past several years [21]. Mass production of Ms&Mb with a good control of size dispersion is undoubtedly possible, and the appropriate processes are employed in the industry; basically, solid glass microspheres are produced by direct heating and melting of glass powders, while glass microbubbles are obtained by adding a blowing agent to the glass powder. 

The sector of solid and hollow glass microspheres has a relevant commercial interest; according to a market research report, the global glass microspheres market is expected to reach $1993.36 million by 2019, with an annual growth around 12.4% [22]. As it could be expected, several patents exist, which cover the subject of glass microsphere and microballoons fabrication, in view of various applications. Table 1 gives a representative, and not exhaustive, list of the US patents on this topic.

### 2.2. Chalcogenide Glass Microspheres

Chalcogenide glasses, namely compounds formed predominately from one or more of the chalcogen elements (Sulfur, Selenium, and Tellurium), are interesting materials in photonics due to their nonlinear properties, photosensitivity, low phonon energy and infrared transparency [23]. Since chalcogenide optical fibers [24] are commercially available, the fabrication method based on the melting of the tip of a fiber can be used in this case as well. A more usual process, however, is to drop the crushed glass through a vertical furnace purged with an inert gas, typically argon [25]. The use of an inert atmosphere is necessary due to the reactive nature of molten chalcogenide glass melting. These solid microspheres find application in biosensing, temperature sensing, lasers and amplifiers [26,27,28]. It is worth mentioning that binary, ternary and quaternary metal-chalcogenide nanocrystals (e.g., CdSe, PbTe, CuInS_2_, Cu_2_ZnSnS_4_ etcetera) are also of interest in the field of renewable energies, to enhance the efficiency of energy conversion devices [29].

### 2.3. Polymer Microspheres

Polyethylene (PE), polystyrene (PS) and polymethylmethacrylate (PMMA) microspheres are among the most common types of polymer microspheres; there are, however, many more polymers and synthesis techniques which can be selected depending on the application. As an example, PS microspheres are typically used in biomedical applications due to their ability to facilitate procedures such as cell sorting. PE microspheres are often used as a permanent or temporary filler, but their high sphericity also makes them suitable for various research application (e.g., microscopy techniques, flow visualization, biomedicine). PMMA microspheres (aka acrylic microspheres) have good biocompatibility which allows the particles to be used in many medical and biochemical applications. All these particles are commercially available; for instance, PE microspheres are available from Cospheric (Santa Barbara, CA, USA) in particle size from 1 μm to 1.7 mm [30] and from Polysciences (Warrington, PA, USA) in sizes from 50 nm to 90 μm [31]; PS microspheres are available from MagSphere (Pasadena, CA, USA) in size ranges from 30 nm to 15 μm [32] and from Microspheres-Nanospheres (Cold Spring, NY, USA) in sizes from 50 nm up to 250 μm [33]; PMMA microspheres (Degradex®) can be obtained from Phosphorex (Hopkinton, PA, USA) in the size range 25 nm to 375 µm [34], and from Microbeads (Skedsmokorset, Norway) in standard sizes of 6, 10, 15, 20, 30 and 40 microns (the cross-inking degree of the beads can be adjusted according to application requirements) [35], while Goodfellow (Huntingdon, UK) offers precision PMMA spheres in two diameters of 1.5 mm and 3.18 mm [36]. In many cases, the spheres are available with a coating, but also with opaque, paramagnetic, fluorescent, and phosphorescent properties.

As their glass counterparts, single polymeric spheres as well are of interest as WGM resonators; some authors have reported the fabrication and characterization of polydimethylsiloxane (PDMS) microspheres [37,38,39]. Single microspheres can be obtained by dipping the tip of an optical fiber into a mixture of PDMS and a curing agent [37]. A larger quantity of PDMS microspheres, with a certain size distribution, can be prepared by mechanical stirring using surfactant solutions [38] or exploiting liquid instabilities [39]. An alternative method, very simple and time-saving, to obtain a high quality polymeric WGM microresonator consists in using a droplet of a commercially available UV-curable adhesive and transferring it onto the tip of an optical (standard or tapered) fiber [40]. On the other side, there exist several polymerization techniques, such as emulsion, dispersion, precipitation and suspension polymerization, which can be used to produce large quantities of polymeric microspheres [41]; many other techniques, such as inkjet printing, electrospraying, and self-assembling processes, may also be adopted for various applications, biology and medicine being one of the most common [42,43,44]. 

The importance of polymer microbubbles in biomedicine is also undoubted, being used especially as contrast agents for medical imaging and as therapeutic delivery devices; their fabrication has been the subject of many R&D investigations [45,46,47,48,49,50,51]. As an example, Table 2 presents a comparison of different methods for preparing polystyrene microbubbles. It appears that the microencapsulation method is the most suitable for preparing PS hollow microspheres in a quite wide range of sizes [47]. Hollow polymer microspheres with different wall materials, however, may need other, more appropriate, methods.

## 3. Applications in the Field of Energy

**Figure 4:**
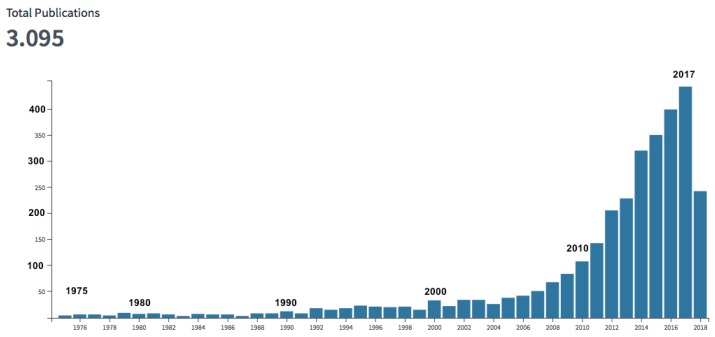
Number of publications with the word “microspher*” in the title and “energy” among the topics. Data from Clarivate Analytics Web of Science; search performed on 21 July 2018.

As anticipated, solid and hollow microspheres have many applications, which depend on the properties of the constituent material and the size, and involve a wide range of technologies. Their use in several different fields has attracted much interest for many years [52,53,54,55,56,57,58,59]. Nowadays, from an industrial point of view, healthcare and biotechnology are the dominant sector, especially due to the development of drug delivery systems [52,55,57]; together with the sector of cosmetics and personal care, it covers more than 50% of the world market. The construction industry, paints and coatings, and automotive are the other relevant industrial application areas [59]. Depending on the application, sometimes ceramic or crystalline microspheres have better properties; glassy Ms&Mb remain, however, the most used components. This is true also in the case of energy applications, an area that has been becoming increasingly important in recent decades. Here, we can categorize the use of Ms&Mb into three sub-areas: energy saving, energy storage, and energy production. A quantitative feeling of the increased interest in the energy applications of microspheres may be obtained by looking at the number of publications: according to Clarivate Analytics Web of Science, the articles containing the word “microspher*” (i.e., microsphere or microspheres or microspherical) in the title add up to almost 46,000 (~18,000 in the last 5 years, and 4143 in 2017). The articles having the words “microspher*”and “energy” in the title are only 213, but those with “microspher*” in the title and “energy” in the topic are over 3000 (over 1700 in the last 5 years and 443 in 2017); in both cases there has been a continuous growth, as one can see in Figure 4, which summarizes the data in a graphical form. It is interesting to note that, by classifying the 3095 articles of the latter database by country/region, it appears that over 50% of the authors are in Asia, only about 12.5% in America and as many in Europe; this classification, however, is far from being accurate, also because more than 20% of the Clarivate records do not contain data in the relevant field.

### 3.1. Energy Saving

**Figure 5:**
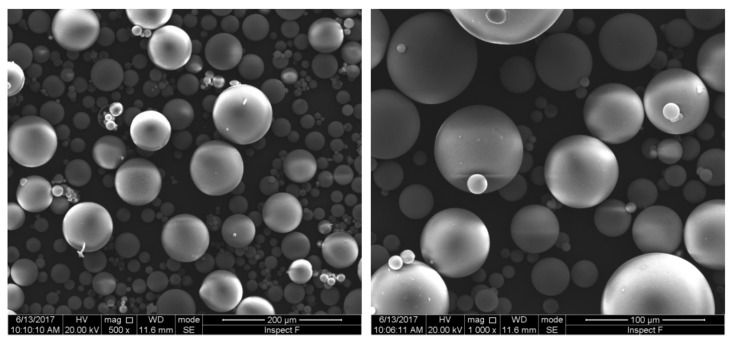
Two SEM (Scanning Electron Microscopy) images of HGM at different magnifications. The size of microbubbles ranges from ~10 μm to ~100 μm, most of them being in the interval 30 to 70 μm. Reproduced from [64] under Creative Commons license.

Hollow glass and polymeric microspheres find wide application in the field of thermal insulation, owing to their distinctive properties, such as high compressive strength, low density, low water absorption, low heat conduction, and high chemical resistance. One of the ways hollow glass microspheres (HGM) help us to reduce energy consumption is their use in oil and gas drilling and extraction operations. In fact, HGM have good rolling characteristics, which can significantly improve the drilling performance; moreover, drilling fluids with HGMs exhibit high temperature resistance, high pressure resistance, stability, and durability, also inducing a longer lifetime of the drilling equipment [60,61]. The energy-saving applications of HGM, however, are particularly relevant in the construction sector, since the residential energy consumption is continuously increasing, especially due to the poor insulation of many buildings and to air conditioning, which in some cases can account for over 50% of the total electricity consumption of the building. A cost-effective solution to reduce this energy waste consists in minimizing the solar heat load and the heat dispersion through the roof and walls by using insulator coating materials that have low thermal conductivity and high infrared radiation reflectivity [62,63]; multiple layer thermal insulation coatings may be the most effective solution [63]. The thermal characteristics of HGM have been the subject of several papers, where different aspects were investigated, such as the mechanism of heat transfer in HGM [64,65] or the effect of inclusion of HGM in different materials [66,67,68,69,70,71,72]. Figure 5 shows a typical scanning electron microscope (SEM) image of soda-lime silicate glass microbubbles fabricated by Sinosteel Maanshan New Material Technology (Maanshan, China) [64]. In the figure, it can be clearly seen that the bubbles are in perfect spherical shape and with a rather broad size distribution.

In the design of insulating structures, it is also important to investigate the long-term durability of the material as a function of different environmental parameters such as water, temperature, and pressure. As an example, Zhang et al. [72] developed a double layer coating composed of an anticorrosive epoxy ester primer and an HGM-containing silicone acrylic topcoat. The HGM size must be properly selected to provide balanced performance on both anticorrosion and heat insulation. An approach to achieve, together with high IR reflection, surface protection from fouling, and therefore longer lifetime, is based on the coating of the HGM by anatase TiO_2_ and a superhydrophobic agent (PFOTES-1H,1H,2H,2H-Perfluorooctyltriethoxysilane) [73]. The utility of including HGM to enhance the thermal and mechanical properties of insulating foams has been proved for a long time [74]; in a recent work, a polysiloxane foam was prepared through foaming and crosslinking processes and reinforced with hollow microspheres, which had been modified with vinyl trimethoxysilane (VMS) to improve the compatibility between the filler and the matrix. The thermal stability and the mechanical properties of the reinforced foam were significantly enhanced: the HGM acted offering many nucleation sites, which was favorable in the formation of a uniform cell morphology, with the only disadvantage that they can easily aggregate in the polymer matrix. The foam with 5% VMS–HGM yielded a minimum thermal conductivity of 0.078 W/mK [75]. In the construction sector, HGM may also be used to partially replace Portland cement in a lightweight foamed concrete: depending on the percentage of HGM, one can obtain a higher compressive strength or a lower thermal conductivity, e.g., going from 0.2507 W/mK of the full cement to 0.2029 W/mK of the foam with 6% soda-lime glass HGM [76].

**Figure 6:**
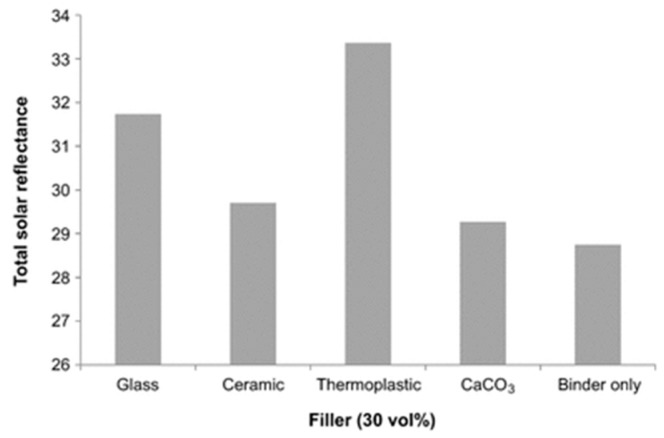
Comparison of the total solar reflectance R_sol_ for similar coatings using different fillers in the same quantity (30 vol%). The value for the binder only is also shown. R_sol_ is calculated by integrating the measured reflectance data in the interval 300 to 2500 nm. Reproduced from [83] under Creative Commons license.

Hollow polymer microspheres (HPM), too, are largely used in insulating materials. As for HGM, surface modification of HPM may be necessary to improve the compatibility of the particles with the matrix; in fact, the low density (25 kg/m^3^) of light HPM fillers produces a heterogeneous dispersion in polymer latex, which in turn may result in poor stability of the insulation coatings. Ye et al. [77] developed a simple process to produce nano-TiO_2_/HPM core-shell composite particles and applied them in external wall thermal insulation coatings. By adding the novel material to a traditional coating and choosing the optimal volume ratio, the thermal conductivity was reduced about nine-fold, reaching 0.1687 W/mK [77]. Thermally expandable polymer microspheres (EPM) are one of the most widely used foaming additives used today; they consist of core/shell particles in which a blowing agent, typically a low boiling hydrocarbon, is encapsulated by a thermoplastic polymer shell [78,79,80]. When heated, the hydrocarbon pressure inside the polymer shell increases while the shell itself softens; thus, EPM expand to a target diameter and maintain that diameter after cooling. Fully expanded, each microsphere may increase up to fivefold its original diameter, with over 100 times increase in volume. Two major suppliers of EPM are AzkoNobel (Amsterdam, The Netherlands) [81] and Kureha (Tokyo, Japan) [82]. Sandin et al. [83] proved that EPM reflect solar radiation over a very broad band (UV, Vis, and NIR) much better than dense fillers, not only in traditional white roof coatings but also in tinted coatings. It is the efficient reflection of near-IR radiation which enables tinted cool roof coatings. Figure 6 presents the calculated total solar reflectance (R_sol_) values for a color-matched blue coating of thickness 600 ± 50 μm which contains 30 vol% of different fillers (namely, CaCO_3_, glass microspheres, ceramic microspheres, and thermoplastic microspheres). The relatively low reflectance values are obviously due to the absorption in the visible due to the blue color; the performance of the thermoplastic (EPM-containing) coating is clearly superior to the other coatings [83].

Another interesting application of HGM has been recently proposed by Zhai et al., who demonstrated efficient day- and night-time radiative cooling by using a novel metamaterial which can be manufactured by a high-throughput, economical roll-to-roll process [84]. The concept of radiative cooling is well known: the energy of a hot body is released via the emission of infrared thermal radiation through the atmospheric window, with the heat being dumped directly to the outer space [85,86,87]. This metamaterial contains SiO_2_ microspheres, with size in the range 4 to 8 μm, randomly distributed in a matrix material of polymethylpentene (TPX) that possesses an excellent solar transmittance. Since the encapsulated silica microspheres, too, have negligible absorption in the solar spectrum, the material is not heated by direct solar irradiance; moreover, it exhibits an infrared emissivity greater than 0.93 across the atmospheric window. When backed by a silver coating, 50 μm thick films showed a noontime radiative cooling power of 93 watts per square meter under direct sunshine, thus allowing to cool objects under direct sunlight with zero energy and water consumption [84].

### 3.2. Energy Storage and Production

In many cases, the technologies for energy production and energy storage are closely interconnected; let us consider here three examples of Ms&Mb use, referring to solar energy, fuel cells, and nuclear energy, respectively.

In the field of solar energy production, glassy microspheres have given only a marginal contribution; an example is represented by a flexible cover glass for solar panels in space applications. The cover glass, patented under the name of Pseudomorphic Glass (PMG) [88], consisted of sphere-like beads typically made of fused silica or ceria-doped borosilicate glasses and diameter of 20–40 μm, embedded in a polymer matrix. The glass can be sprayed onto the solar cells or can be manufactured in the form of microsheets that adhere to the solar cells; it proved to increase the efficiency and the UV transmittance with respect to conventional materials. Further, a multi-layer hybrid PMG cover glass using a thin top layer of ceria-doped borosilicate beads and a bottom layer of fused silica beads guarantees enhanced UV protection and a broadened spectral transmission bandwidth. Another patent was claiming to enhance the properties of an encapsulation adhesive film for a solar cell module; the film was made by mixing transparent microspheres (either polymeric or glassy or ceramic), with an average diameter in the range of 0.1 to 50 μm, together with an adhesive film [89]. The scattering and multiple reflections by the microspheres could improve light harvesting by the solar cell, thus increasing the electrical power generation. An increase in light trapping was also demonstrated in a periodic structure of microspheres deposited using a self-assembly method on the surface of a GaAs solar cell: an increase of about 25% in the conversion power efficiency of the cell was measured when using microspheres with size of 1 μm [90]. It may be interesting to note, even if the device is millimetric and not glassy, that the spherical shape has been adopted to make full solar cells, too: the Japanese company Kyosemi developed Sphelar, a spherical solar-cell technology that captures sunlight in three dimensions [91]. Single spherical cells are produced by dropping molten silicon into a tube, where the silicon droplets become rounded by surface tension during the free fall. Then, a proper process, which includes phosphorus diffusion and deposition of thin electrodes, creates a spherical p–n junction between the inner and outer parts of the crystalline sphere, with diameter of 1–2 mm. These tiny spherical Si solar cells can be incorporated into a variety of transparent materials, creating modules capable of covering a wide range of voltages [91].

In recent years, environment protection has pushed the search for vehicles having less harmful impacts to the environment than internal combustion engine vehicles running on gasoline or diesel. One of the best solutions appears to be that of electric vehicles, which, in turn, are expected to have great advantages from the use of fuel cells [92]; electric vehicles powered by fuel cells can travel for 500 km or more on a tankful of fuel, and—this is the best point—they can be refilled, as with a conventional car, in a matter of minutes rather than hours, unlike battery vehicles. Hydrogen storage in a small volume and light weight, however, is a significant challenge for the development and viability of hydrogen-powered vehicles [93,94]. Here it is where hollow glass microspheres can prove their capabilities: one can exploit the diffusion of hydrogen through the thin wall of an HGM at elevated temperatures and pressures, and then let the gas to be trapped upon cooling to room temperature. HGM with diameter in the range 1 to 100 μm, density between 1.0 and 2.0 gm/cc, and porous-wall structure with wall openings 1 to 100 nm represent a promising material for hydrogen storage, as demonstrated in recent papers and patents that have shown progress in the preparation and use of HGM for this application [95,96,97,98,99]. The storage of hydrogen at pressures up to 100MPa inside an HGM is possible due to the low diffusivity of hydrogen at room temperature; later, to release it, it is necessary to reheat the microspheres. However, a limitation of HGM has been the poor thermal conductivity, which implies unsuitably low release rates of hydrogen gas; to overcome this problem, a proposed solution consisted of doping the glass with transition metals. As an example, cobalt loaded HGM, prepared by mixing cobalt nitrate hexahydrate with the glass powder and using an air-acetylene flame for melting the particles and producing the microspheres, showed an increase of thermal conductivity from 0.072 to 0.198 W/mK when the cobalt loading increased from 0 to 10 wt.% [97]. Hydrogen adsorption capacity, however, has a maximum for cobalt loading at 2 wt.%; beyond 2%, the storage capacity is said to decrease due to the closure of the pores by the uneven deposition of CoO on the surface of the microspheres [97].

Proper strategies must be developed for applications in which rapid storage/release of stored gas is required: one of them is based on the photo-induced outgassing, in which an infrared light lamp is used to accelerate the release rate in comparison with furnace heating. To enhance this outgassing, one must follow the same approach used to increase the thermal conductivity, i.e., doping the glass with “optically active” dopants such as iron, nickel, and cobalt. Rapp and Shelby [100] worked on various borosilicate Corning glasses and various dopants and found a good response by the 0.5 wt.% Fe_3_O_4_-doped 7070 borosilicate glass; in this case, the amount of hydrogen released was proportional to the lamp intensity. Moreover, the reaction of hydrogen with the iron-doped glass increases the Fe^2+^/Fe^3+^ ratio, which promotes infrared absorption and thus further enhances the hydrogen yield obtained from photo-induced outgassing. More recently, Shetty et al. [95] developed a facile flame spraying method for producing cobalt-doped HGM using recycled amber glass frit coated by a transition metal salt. They found that doping with 3 wt.% CoO was more effective at photo-induced outgassing than 1.5% wt.% CoO. In addition, a model was developed to estimate the conditions needed to produce HGM with engineered geometric properties, i.e., wall thickness and aspect ratio (diameter divided by the wall thickness).

It may also be worth to mention that crystalline hollow microspheres (e.g., vanadium pentoxide or multishelled TiO_2_ and NiO microspheres) may play an important role in electrical energy storage, being used as safe, inexpensive anode materials for lithium ion batteries [101,102] and supercapacitors [103], respectively.

The property of HGM to be permeable to gases and their ability to safely store such high-pressure gases make them attractive for a very important application in the field of nuclear energy. As early as in late 1950s, a group at Lawrence Livermore National Laboratory in the USA was studying how to fill capsules with a mixture of deuterium and tritium (DT) to be compressed until reaching nuclear fusion. In 1972, Nuckolls et al. published a paper in *Nature* showing that the efficient laser thermonuclear burn of small pellets of DT was feasible, opening the way to the development of fusion power reactors [104]. Let us refer to a few recent publications to have an idea of the basic challenges and state of art of the research on inertial confinement fusion (ICF) [105,106,107,108]. Since the 1970s, much attention has been focused on the preparation of the nuclear fusion targets filled with hydrogen or its isotopes, since the success of any laser-fusion system depends critically on the low-cost production of suitable fuel capsules that satisfy the overall requirements. Hollow glass or polymer microspheres, with diameters in the range of approximately 50 to 500 μm and wall thickness between 1 and 20 μm appeared to be very good candidates; many papers and patents have been published concerning the fabrication and/or the filling of these microcapsules, and a few of them are cited here [109,110,111,112,113,114,115,116,117,118,119,120].

**Figure 7:**
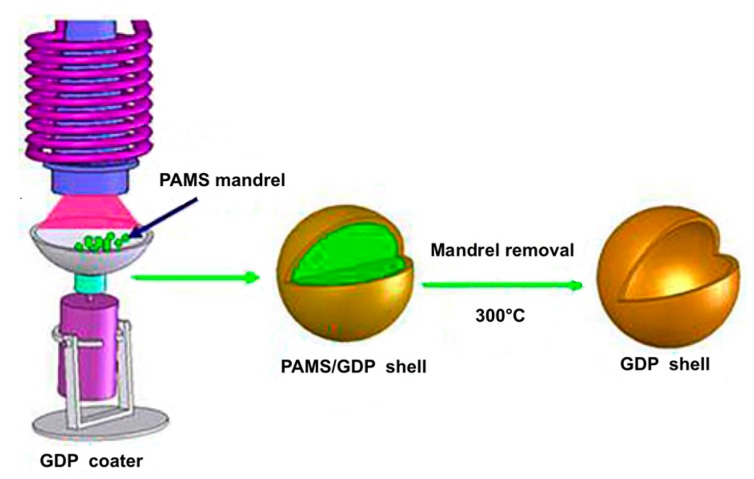
Schematic process for the fabrication of GDP shell. (GDP-glow discharge polymer; PAMS-poly α-methylstyrene). Reproduced with modifications from [122] under Creative Commons license.

According to a recent review on the development of target fabrication for laser-driven ICF at the Research Center of Laser Fusion (RCLF) in China [121], glow discharge polymer (GDP), glass, and polystyrene (PS) hollow microspheres are among the candidates for the ultimate ignition. Let us just refer to the first type: GDP microballoons can be produced by inductive coupled plasma enhanced chemical vapor deposition (ICP-CVD), a method that permits the deposition of high quality dielectric films at low temperature with low damage. Trans-2-butene and H_2_ were utilized as the working gases, and the GDP coating was deposited on mandrels made from poly α-methylstyrene (PAMS). A conical quartz tube used as the plasma generator allowed a fast growth rate of ~1.5 m/h; to get homogeneous coating of the mandrels, they were made to roll randomly inside a special designed glass pan. To produce single layered GDP microballoons after the coating deposition, the double layered PAMS/GDP spheres were annealed in vacuum or Ar atmosphere at 300 °C for more than 24 h, so to pyrolyze the mandrel. Typically, 8 µm thick single layered GDP shells with diameter of 450–540 µm were manufactured. Figure 7 summarizes the process [122]. It can be noted that the basic PAMS/GDP process for production of ICF target mandrels had been already tested in 1997 [118].

The possibility of encapsulating several fuel-filled spheres in a low-density foam was also investigated and patented [123,124]; such a foam was requested to have a cell size smaller than 2 μm, a density of about 0.1 × l0^3^ kg/m^2^, and a chemical composition of low average atomic number.

With the increase of the available laser power, the design of the targets has become increasingly complex, and several structured target configurations have been reported, often comprising a multilayer structure [125]. One of the layers usually is a low-atomic-number polymer coating that must ablate as the laser pulse irradiates its surface: the ablation imparts a reaction force to the core material, causing the fuel within to be compressed to high density. The polymeric layers must have a predetermined thickness and a surface finish smoother than 0.1 μm and they must conform perfectly to the glass sphere; the deposition technique is therefore very important [114,126,127]. In recent years, laser-fusion programs seem to have moved to consider larger fuel capsules [121,128]; viable ICF targets are represented by spherical shells with diameter 0.5 to 4 mm, wall thickness 50–100 μm, low density (~250 mg/cm^3^), with interconnected voids (each <1 μm diameter), with extreme sphericity (>99.9%, <50 nm roughness variation), and a high degree of concentricity (>99.0%) [129]. Fabricating these pellets with so stringent specifications is a big technical challenge, and even more challenging is the fact that they should be produced at massive scale. In fact, a reliable and economic fuel supply is essential for the viability of future ICF power plants, where the problem nowadays is not the pellet’s content, namely DT, but the container itself, namely the spherical capsule. It is estimated that six targets per second, or about 500,000/day, with a cost below 0.25 $/target (orders of magnitude less than current costs), will be required for a power plant with nominal electric output of 1000 MW [130]. The efforts to improve the quality of the targets [131] and to develop the possibility of their large-scale production have made significant progress in recent years. As an example, Li et al. developed a continuous and scalable process for the fabrication of polymer capsules using droplet microfluidics, thus demonstrating that, even with the many remaining limitations, channel-based droplet microfluidics technology has the potential of being applied to ICF target fabrication [129].

## 4. Conclusions

Scientific and commercial applications of solid and hollow glass and polymer microspheres have been continuously growing in recent decades, in parallel with the advances in their fabrication with high quality and large batches. On the other hand, single microspheres have also gained much attention for their potential as ultra-high quality-factor WGM optical microresonators [2]. One of the advantages of glassy microspheres is that they can be easily doped with chemical elements and compounds to increase their functionality; moreover, they can be made porous or hollow, allowing for encapsulation of other chemical or biomedically relevant components. All these properties open the way to develop microlasers, microsensors, biolabeling or drug-delivery bullets or even to study matter-radiation interactions at the very high power density made possible by the strong light confinement of WGMs [132,133,134,135,136]. Industrial applications are also mature, and it appears that there is a constant or increasing demand from the healthcare and construction (e.g., paints and coatings) sector. Much of the ongoing research relates to the advances in the polymer industry because the possibility of changing the molecular properties (and hence the chemical and physical properties) permits to conceive new applications of polymeric microspheres.

In the field of energy, glassy microspheres (and nanospheres) may offer effective solutions to some of the problems that arise in the advanced technologies of energy generation. At a lower technological level, they can be used for thermal insulation, or as proppants for shale oil and gas recovery, components for solar cells, or anodes for electrical batteries. In all these cases, the synthesis process of organic or inorganic microspheres does not present significant difficulties and the width of size distribution of the produced particles is not a critical factor; current research is aimed at optimizing the choice of the mean size and the usage protocol for the specific application considered. There is, however, a research topic of growing interest where the size of the micrometric and sub-micrometric spheres must be controlled: it is the case of photonic crystal structures and arrays of resonators, whose properties may be exploited to improve light harvesting in solar cells [137,138,139,140]. A deeper study of the self-organization processes of colloidal particles and of the process parameters would be important to optimize the design and implementation of photonic crystal structures [137,139].

At a higher technological level, hollow glass and polymer microspheres have a winning potential in two areas, namely hydrogen storage and ICF, which are of great importance for decreasing today’s CO_2_ and particles’ emissions and taking care of the environment. In both applications, the peculiar properties of hollow porous microspheres can be fully exploited. In particular, the fabrication of the shell targets used in ICF experiments is a challenging project that involves different disciplines and advanced material preparation and manufacture process.

Many more challenges and difficulties can be seen ahead, e.g., to develop reliable, very high quality and economic mass-production technologies, but the horizon appears sunny for these microdevices.

## Figures and Tables

**Table 1:** List of United States Patents referring to the fabrication process of glass microspheres. The superscripts s,h indicate solid and hollow microspheres, respectively.

Inventors	US Patent N.	Year	Title
Veatch, F.; Alford, H.E.; Croft, R.D.	2,978,339	1961	Method of producing hollow glass spheres ^h^
Beck, W.R.; O’Brien, D.L.	3,365,315	1968	Glass bubbles prepared by reheating solid glass particles
Tung, C.F.; Laird, J.A.	3,946,130	1976	Transparent glass microspheres and products made therefrom ^s^
Garnier, P.; Abriou, D.; Coquillon, M.	4,661,137	1987	Process for producing glass microspheres ^h^
Block, J.; Lau, J.W.; Rice, R.W.; Colageo, A.J.	5,176,732	1993	Method for making low sodium hollow glass microspheres
Arai, K.; Yamada. K.; Hirano H., Satoh M.	5,849,055	1998	Process for producing inorganic microspheres ^s,h^
Henderson, T.M.; Wedding D.K.	6,919,685	2001	Microsphere ^h^
Yamada, K.; Hirano, H.; Kusaka, M.; Tanaka, M.	0043996 (Application Publication #)	2001	Hollow aluminosilicate glass microspheres and process for their production ^h^
Kirkland, J.J.; Langlois, T.J.; Wang, O.	6,482,324	2002	Porous silica microsphere scavengers ^s^
Tanaka, M.; Hirano, H.; Yamada, K.	6,531,222	2003	Fine hollow glass sphere and method for preparing the same ^h^
Lipinska-Kalita, K.E.; Hemmers, O.A.	8,663,429	2014	Hollow glass microsphere candidates for reversible hydrogen storage, particularly for vehicular applications ^h^

**Table 2:** Comparison of different methods for preparing polystyrene hollow microspheres. Reproduced with modifications from [47] under Creative Commons license.

Properties	Liquid Droplet Method	Dried-Gel Droplet Method	Self-Assembly Method	Micro-Encapsulation Method	Emulsion Polymerization Method	Template Method
Equipment cost	High	High	Low	Low	Low	Low
Operation cost	High	High	Low	Low	Low	High
Micromanipulation	Yes	Yes	Yes	No	No	Yes
Batch production	Able	Able	Able	Able	Able	Able
Multiwalled product	No	No	Able	Able	Able	Able
Microsphere diameter, μm	500 ÷ 1500	500 ÷ 1500	≤0.5	50 ÷ 700	≤20	≤5
Sphericity, %	≥97	≥99	≥99	≥99	≥99	≥99
Concentricity, %	≥90	≤90	≥99	≥98	≥98	≥99
Surface roughness, nm	<200	<200	<10	<300	<10	<5
